# Integrative QTL mapping and candidate gene analysis for main stem node number in soybean

**DOI:** 10.1186/s12870-025-06457-2

**Published:** 2025-04-03

**Authors:** Bire Zha, Chunlei Zhang, Rongqiang Yuan, Kezhen Zhao, Jianqiang Sun, Xiulin Liu, Xueyang Wang, Fengyi Zhang, Bixian Zhang, Sobhi F. Lamlom, Honglei Ren, Lijuan Qiu

**Affiliations:** 1 Soybean Research Institute of Heilongjiang Academy of Agriculture Sciences, Harbin, 150086 China; 2 College of Modern Agriculture and Ecological, Environment of Heilongjiang University, Harbin, Heilongjiang China; 3 College of Agronomy, Shenyang Agricultural University, Shenyang, Liaoning China; 4 Institute of Crop Sciences, Mlinistry of Agriculture and Rural Affairs, Key Laboratory of Crop Gene Resource and Germplasm Enhancement, Chinese Academy of Agricultural Sciences, National Key Facility for Crop Gene Resources and Genetic improvement (NFCRl), Ministry of Agriculture and Rural Affairs, Beijing, 100081 China; 5 Institute of Biotechnology of Heilongjiang Academy of Agricultural Sciences, Harbin, China; 6 Workstation of Science and Technique for Post-doctoral in Sugar Beet Institute, Heilongjiang University, 74 Xuefu Road, Harbin, 150000 Heilongjiang China; 7 Plant Production Department, Faculty of Agriculture Saba Basha, Alexandria University, Alexandria, 21531 Egypt

**Keywords:** Main stem node number (MSNN), KASP marker, SLAF sequencing, Soybean, QTL mapping

## Abstract

Main stem node number (MSNN) is a key yield-related quantitative trait that directly affects the number of branches and seeds per soybean plant. In this study, a QTL mapping using SLAF sequencing and candidate gene analyses were used to determine the detailed genetic basis of MSNN across a diverse set of soybean line. This study investigated the variation characteristics of MSNN in 325 recombinant inbred lines (RILs) obtained from the hybridization of Qihuang 34 and Dongsheng 16. The phenotypic analysis revealed prominent transgressive segregation and continuous variation in MSNN, with a normal distribution observed for MSNN in the RIL population. A genetic map including 6297 SLAF markers was developed which spanned 2945.26 cM, with an average genetic distance of 0.47 cM between adjacent markers. QTL mapping identified five significant QTLs associated with MSNN, were located on chromosomes 6 (*qMSNN6.1*), 17 (*qMSNN17.1*), 18 (*qMSNN18.1*), and 19 (*qMSNN19.1* and *qMSNN19.2*) with LOD values ranging from 3.89 to 37.92, explaining 3.46–43.56% of the phenotypic variance. Among the five QTLs, *qMSNN19.2 recorded t*he highest LOD value, 37.92, indicated a stable environment QTL explaining 43.56% of the variance. Candidate gene mining revealed 64 genes located in the QTL *qMSNN19.2*, with selections made based on biological processes like regulation of stem cell division and plant hormone signaling. Additionally, specific SNP variations in candidate genes were identified for KASP marker development, offering potential targets for enhancing soybean MSNN traits. The findings of this study can assist the soybean breeding programs for developing cultivars with desirable MSNN.

**Supplementary Information:**

The online version contains supplementary material available at 10.1186/s12870-025-06457-2.

## Introduction

Soybean (*Glycine max* (L.) Merr.) is a globally significant crop, serving as a key source of protein and oil for human consumption, animal feed, and various industrial applications [1, 2]. As global demand for soybeans rises due to population growth and changing dietary habits, enhancing soybean production and adaptability has become increasingly critical [3, 4]. China, the world’s largest soybean consumer, heavily depends on imports to meet its commercial needs [5, 6]. Despite decades of efforts to increase soybean yields in China, progress has been limited, underscoring the urgent need to boost domestic production and achieve self-sufficiency in soybean cultivation [7, 8]. To address this, breeders are targeting various yield-related traits, with the stem node being a key focus due to its influence on plant architecture, adaptability, and yield potential [9–11].

MSNN plays a significant role in determining plant architecture, biomass accumulation, and overall yield potential [12]. The arrangement of nodes in soybean plants is particularly important for photosynthesis and nutrient distribution, both of which directly affect seed yield [9, 13]. MSNN influences plant height and branching, which in turn affect light interception and photosynthetic efficiency [10]. Additionally, node number impacts the distribution of reproductive structures such as pods and seeds, thereby directly influencing yield outcomes [14, 15]. Understanding the genetic basis of node number variation in soybean populations is essential for developing high-yielding cultivars [15, 16]. MSNN is a typical quantitative trait, and the interaction of the genotype and the environment complicates the study on genetic basis. Therefore, molecular markers are widely used to locate quantitative trait loci (QTL) to reveal the molecular mechanism of MSNN in soybean yield [17, 18].

The investigation of genetic variation associated with MSNN has garnered significant interest in QTL mapping [17, 19]. Utilizing diverse mapping methodologies, researchers have pinpointed many QTLs linked to MSNN, elucidating the intricate genetic framework regulating this characteristic. The identification of these QTLs enables breeders to more efficiently select for desirable phenotypes via marker-assisted selection, hence promoting the production of enhanced soybean varieties suited to diverse environmental circumstances and management approaches. Currently, Soybase (https://www.soybase.org/) has documented 38 QTL for MSNN identified via genetic linkage analysis, corroborated by research from [20–24]. Single nucleotide polymorphisms (SNPs) are crucial for constructing high-density genetic linkage maps and mapping QTLs [25–27]. The SLAF-seq (specific-locus amplified fragment sequencing) method offers a solution by enabling the rapid development of SNP markers after constructing a SLAF-seq library [28, 29]. This technology has been successfully used to create high-density genetic maps for various species due to its high throughput, accuracy, cost-effectiveness, and rapid turnaround [30–32]. SLAF markers have become a powerful tool for genetic analysis in plants due to their high abundance, even distribution across genomes, and ability to avoid repetitive sequences. These properties make SLAF markers highly effective for constructing high-density genetic maps and identifying quantitative trait loci (QTLs). SLAF-seq has been successfully applied in genetic studies of various crops [25, 27, 28, 32–34]. Since the first high-density genetic map was constructed using SLAF-seq [28], several high-density maps have been reported. For example, Qi et al. [33] developed a genetic map containing 5,308 markers with a total length of 2,655.68 cM using a recombinant inbred line (RIL) population derived from a cross between ‘Charleston’ and ‘Dongnong594’ [33]. Similarly, Li et al. [35] constructed a high-density genetic map using an F_5:8_ population of 110 RILs from a cross between ‘Luheidou2’ and ‘Nanhuizao’. This map was used to identify QTLs associated with isoflavone content and fatty acid composition in soybean. In another study, Zhang et al. [36] reported 20 QTLs associated with phosphorus efficiency-related traits based on a high-density map constructed using SLAF-seq [36]. Additionally, Cao et al. [37] mapped QTLs related to plant height and flowering time using a genetic map constructed by SLAF-seq. This study utilized a population of 236 RILs derived from a cross between two summer planting varieties, ‘ZXD’ and ‘NN1138–2’.

This study focuses on the genetic mapping of QTLs associated with MSNN in soybean, utilizing a RIL population derived from a cross between Dongsheng 16 and Qihuang 34 (F_2 − 6_). The primary objectives include identifying QTLs linked to MSNN, uncovering candidate genes involved in this trait, and assessing the reliability and accuracy of Kompetitive Allele-Specific PCR (KASP) as a genotyping tool for QTL mapping. By employing a high-density genetic linkage map constructed through Specific-Locus Amplified Fragment Sequencing (SLAF-seq), the study aims to delineate candidate genomic intervals harboring major loci associated with MSNN.

## Materials and methods

### Soybean populations assessments

The recombinant inbred lines (RILs) of soybean were developed by crossing two parental lines: ‘Qihuang 34’, which exhibits a highest MSNN, and ‘Dongsheng 16’, which has a lowest MSNN. The two parental lines used in this study, ‘Qihuang 34’ and ‘Dongsheng 16’, were provided by Prof. Lijuan Qiu from the Institute of Crop Sciences, Chinese Academy of Agricultural Sciences (CAAS), which is part of the National Key Facility for Crop Gene Resources and Genetic Improvement (NFCRI). A population of 325 F_2:6_ individuals was generated using the single seed descent method. In 2023, the parental lines and RIL population were cultivated at the Harbin Experimental Farm, while in 2024, they were grown at the Yazhou District Experimental Farm in Sanya. Both parents and the RILs were arranged in a randomized block design with three replications. Each plot consisted of three rows, with rows measuring 3.0 m in length, spaced 35 cm apart, and plants spaced 10 cm apart within rows. The field was watered with 120 m³ of water before planting, and on April 30th, two seeds were manually scattered into each hole for sowing. If both seeds germinated, one plant was removed after the development of the second set of trifoliate leaves. Post-emergence, field management followed standard local agricultural practices. For phenotypic evaluation, ten mature plants from the middle row of each plot were randomly selected to measure MSNN before harvest. MSNN was recorded as the number of nodes from the cotyledonary node to the apex of the main stem. The average MSNN value from the three replications was used for further analysis. Phenotypic data analysis was conducted using SPSS statistical software (version 20.0). Chi-square (χ^2^) tests was applied to examine the segregation patterns of the underlying genetic factors in the population, not to categorize the MSNN phenotype using the formula:χ^2^=∑(O − E)^2^/E.

where: O*O* = Observed frequency, E*E* = Expected frequency.

### DNA extraction, construction and genotyping of SLAF libraries

Fresh leaves from the two parents and all 325 RILs were collected into centrifuge tubes, frozen in liquid nitrogen, ground in a tissue grinder, and then stored at -80 °C. Total genomic DNA was extracted from each leaf sample using a modified CTAB method [38]. The quality and concentration of the extracted DNA were determined by 1% agarose gel electrophoresis and a spectrophotometer (UV-Vis Spectrophotometer Q5000). Individual SLAF libraries of 325 RILs and their parents were constructed and sequenced. A combination of Rsa I and Hae III restriction endonucleases was selected to digest genomic DNA. The SLAF markers of 325 RILs were grouped and genotyped using the method of Sun et al. [28]. The high-density molecular tags developed for the RIL population were used to construct a genetic map using HighMap software [39].

### QTL mapping for MSNN in soybean

QTL IciMapping V4.2 software was used for QTL detection based on the phenotypes in the two environments. The complete interval mapping method (ICIM-ADD) was used, the scanning step was set to 1 cM, the stepwise regression marker entry probability was 0.001, and LOD = 3.0 was used as the threshold to detect the existence of a putative QTL.

### Candidate gene prediction

The physical positions of the markers at both ends of the interval were compared with the soybean reference genome (Glyma.Wm82.a2.v1) to find candidate genes in the interval and extract gene numbers and gene annotations. Combined with SoyOmics (SoyOmics - CNCB-NGDC), the specific expression of candidate genes in different tissues and enrichment analysis were analyzed to preliminarily determine the key candidate genes.

### Relative expression analysis

Quantitative real-time PCR (qRT‒PCR) validation was conducted on six candidate genes. Stem samples from the two soybean cultivars at two vegetative stage (V1, and V2) were collected in triplicate. Total RNA was extracted from these samples using an RNA isolation reagent (Thermo Fisher Scientific, USA). First-strand cDNA was synthesized with TransScript® One-Step gDNA Removal and cDNA Synthesis SuperMix (Transgen, China). Gene-specific primers for qPCR were designed using the Primer3 website (Table S1), with the soybean GmActin gene (Glyma.18G290800) serving as the internal reference gene. The qRT‒PCR reactions were conducted using the CFX96 Touch Real-Time PCR Detection System (Bio-Rad, USA) with ChamQ SYBR qPCR Master Mix (Vazyme, China). For gene expression, three independent samples were collected from different points in the stem within each block for each cultivar. These three samples were pooled to create one representative biological replicate, resulting in three biological replicates per cultivar (one pooled sample per block). This composite sampling strategy, covering nine total sampling points per cultivar across the field (three sampling points × three blocks), was implemented to account for field variation while maintaining statistical power.

### Kompetitive allele-specific PCR development

To genotype using KASP markers, three specific primer sets were employed. These primers were designed using Primer-Blast, a tool on the NCBI website. Polymerase chain reaction (PCR) was performed using KASP V4.0 Mastermix according to the manufacturer’s instructions. The amplified DNA was then analyzed via quantitive real-time PCR using the ABI QuantStudio 6 Pro. KASP primers were designed using PolyMarker, which identified single nucleotide polymorphisms (SNPs). The primers were then synthesized and included two allele-specific forward primers and one common reverse primer (Table S2). For each KASP assay, a mix containing the primers and 2X Reaction Mix was prepared and added to 384-well PCR plates with genomic DNA. The PCR program consisted of an initial 15-minute denaturation at 94 °C, followed by 10 touchdown cycles where the temperature decreased by 0.6 °C per cycle, and then 26 cycles of denaturation and annealing at 94 and 55 °C, respectively. The PCR reactions were conducted in a Hydrocycler 16, and fluorescence detection was performed using a PHERAstar microplate reader. The data was analyzed using KlusterCaller software.

## Results

### Phenotypic variation of MSNN in parents and RIL population

**Fig. 1:**
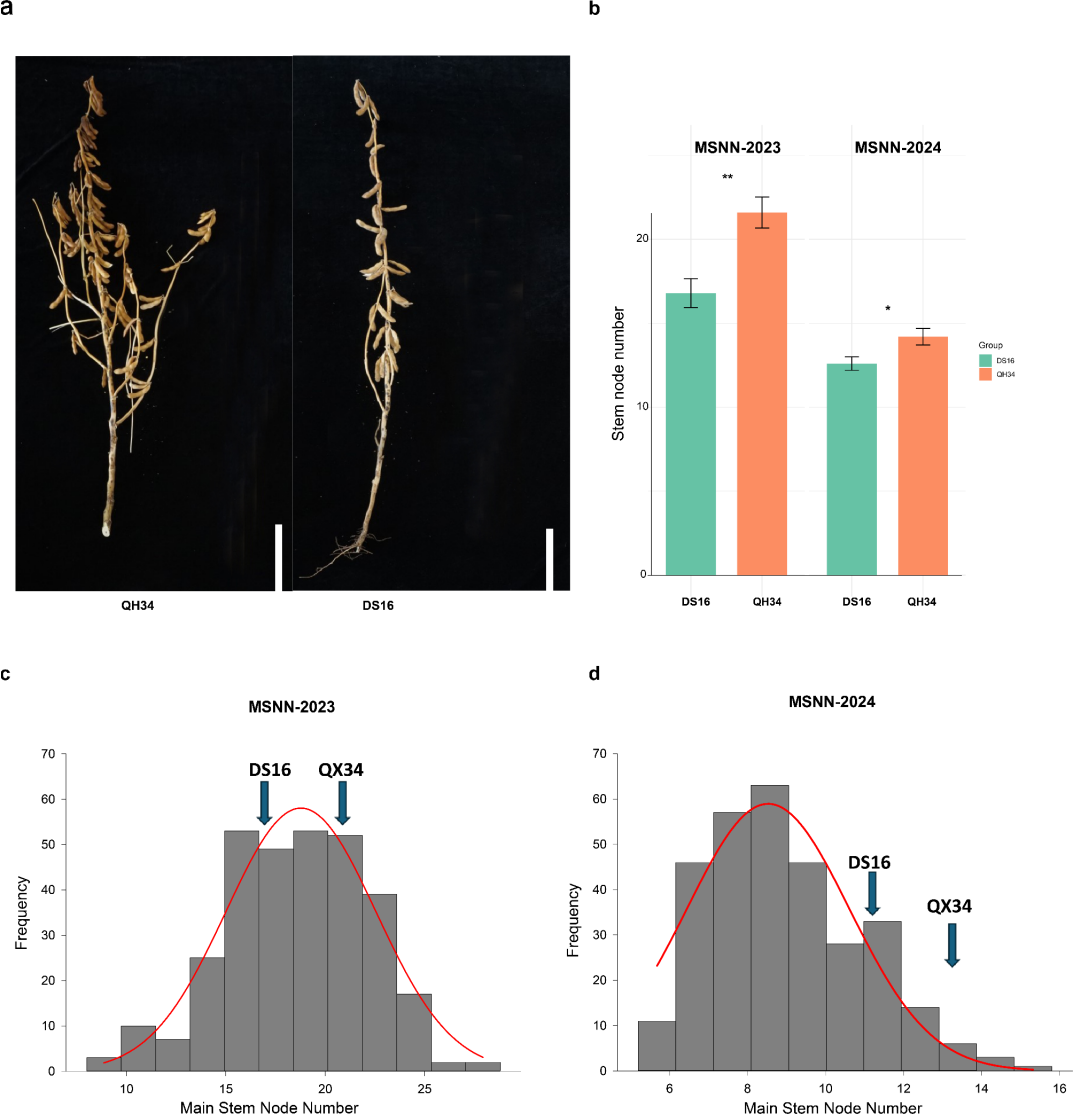
Phenotypic characterization of MSNN in ‘Qihuang 34’ and ‘Dongsheng 16’ (**a**), MSNN morphology of two parents ‘Qihuang 34’ and ‘Dongsheng 16’. (**b**), Comparisons of MSNN between ‘Qihuang 34’ and ‘Dongsheng 16’. Student’s t-test was used to generate the *P* values. (**c**, and **d**) The frequency distribution of the soybean MSNN in the RIL population in 2023 and 2024 seasons

Significant phenotypic variation in MSNN was observed between the parental lines ‘Qihuang 34’ and ‘Dongsheng 16’ (Fig. 1a, b). Phenotypic evaluation of the RIL population over two growing seasons (2023 and 2024) revealed substantial variation in MSNN. In 2023, MSNN values ranged from 8 to 28.8, with a mean of 18.57 ± 3.61. The 2024 season showed a range of 5.2 to 15.8 nodes, with a mean of 8.98 ± 1.94 (Table 1). The coefficient of variation remained relatively stable across both years (0.23 and 0.22 for 2023 and 2024, respectively), suggesting consistent genetic control despite environmental influences. The frequency distribution of MSNN in the RIL population displayed a continuous pattern in both years (Fig. 1c, d), characteristic of quantitative inheritance. The trait showed slight negative skewness (-0.2031) in 2023 and positive skewness (0.524) in 2024. Kurtosis values were near normal in both years (-0.0508 and − 0.1229 for 2023 and 2024, respectively), indicating a typical quantitative trait distribution. The substantial difference in mean MSNN values between years (18.57 vs. 8.98) suggests significant environmental influence on trait expression, while the consistent CV values indicate reliable genetic control. A Chi-square (χ²) test was conducted to evaluate the segregation ratio of the observed population against the expected 3:1 Mendelian ratio. The calculated Chi-square value was 12.345, with 4 degrees of freedom, and the corresponding *p*-value was 0.015. This result indicates that the observed segregation ratio significantly deviates from the expected 3:1 Mendelian ratio at the 5% significance level (*p* < 0.05). Such a deviation suggests that the MSNN trait may be influenced by factors beyond simple Mendelian inheritance, such as genetic linkage, environmental effects, or the involvement of multiple genes.

**Table 1:** Statistical analysis of phenotype of MSNN

Year	Max	Min	Mean	SD	Variance	Skewness	Kurtosis	CV	χ²
2023	8	28.8	18.57	3.61	13.07	-0.2031	-0.0508	0.23	12.345
2024	5.2	15.8	8.98	1.94	3.77	0.524	-0.1229	0.22	

### SLAF- sequence and genotype of parental lines and RILs

**Fig. 2:**
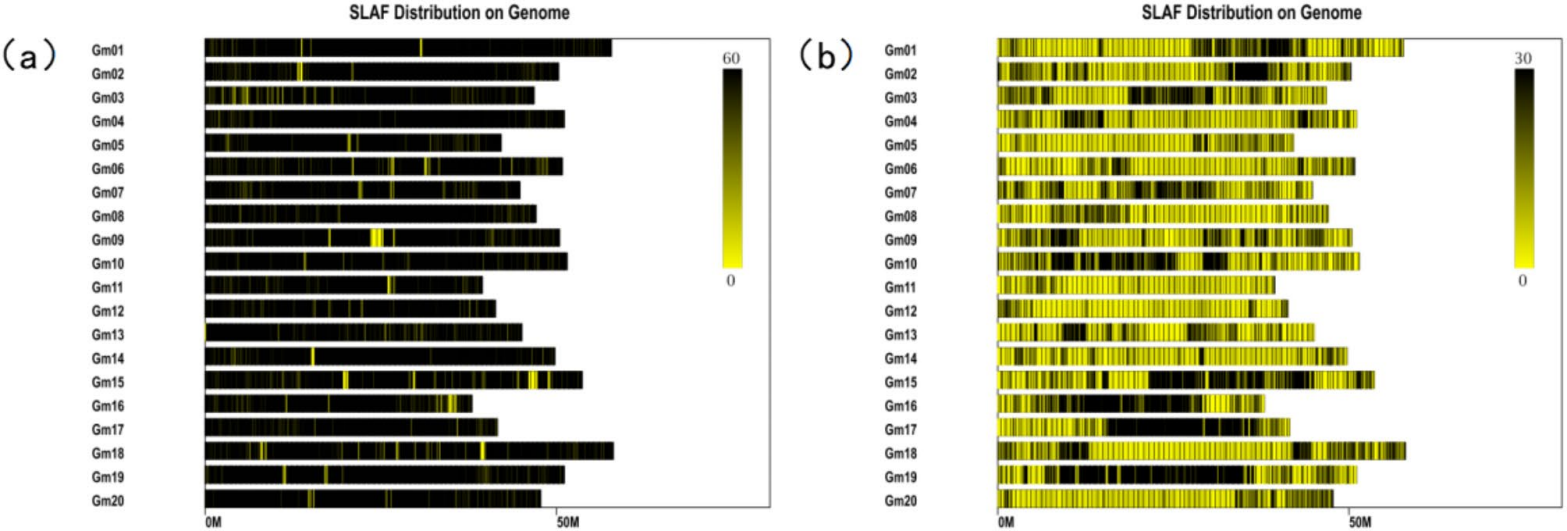
SLAF tag and polymorphism Distribution of SLAF tag. (**a**) shows the distribution of SLAF markers, and (**b**) shows the polymorphic SLAF markers (in M units). Note: The abscissa is the length of chromosome, and each yellow stripe represents a chromosome. The genome is divided into 1 M units, and different colors indicate the number of SLAF. The darker the color, the more SLAF. The darker areas in the figure are the concentrated distribution of SLAF markers. Note: The horizontal axis is the length of the chromosome, and each yellow stripe represents a chromosome

The sequencing depth of Dongsheng 16 was 13.17 x, the sequencing depth of Qihuang 34 was 12.77 x, the average sequencing depth of parents was 12.97 x, and the average sequencing depth of offspring was 10.05 x. A total of 627,099 SLAF tags were obtained. Among them, there were 98,571 polymorphic SLAF tags, and the polymorphism ratio reached 15.72%. The SLAF tags were located on the reference genome using BWA software (https://nchc.dl.sourceforge.net/project/bio-bwa/bwa-0.7.15.tar.bz2), and the chromosome distribution map of SLAF tags (Fig. 2a) and polymorphic SLAF tags (Fig. 2b) were drawn according to the distribution of SLAF tags on chromosomes.

### Polymorphic SLAF tag encoding and screening of mapping SLAF markers

To facilitate subsequent genetic analysis, all polymorphic SLAF markers need to be genotyped. The genotype encoding rule is the common 2-allele encoding rule in genetics. This study is a RIL segregation population, so aa x bb type polymorphic markers are selected, and the paternal genotype is aa, the maternal genotype is bb, and the offspring genotype is aa (paternal type), bb (maternal type), ab (heterozygous type), -- (missing type). Based on the results of parental genotype detection, sites with missing parental information are filtered out. According to the genotype encoding rules in the above table, the 98,571 polymorphic SLAF markers obtained in this study are typed, and 35,174 tags are successfully encoded. To ensure the quality of the genetic map, the polymorphic SLAF markers are filtered to obtain 6,717 SLAF markers that can be used for mapping. The distribution on the chromosome is shown in (Table S3).

### Construction of high-density genetic map

Based on the reference genome, a total of 6717 polymorphic markers were located on 20 chromosomes. After filtering out the markers with MLOD values ​​less than 3 with the SLAF markers, a total of 6297 markers were mapped and located as markers. The mapping rate was 93.75%. Each chromosome is a linkage group. The linear arrangement of markers within the linkage group was obtained by HighMap software analysis, and the genetic distance between adjacent markers was estimated. Finally, a high-density genetic map with a total map distance of 2945.26 cM was obtained (Fig. 1). First, to test the quality of soybean high-density genetic map, the basic information of the number of markers, total map distance, average map distance, maximum gap and gap < 5 cM ratio of each linkage group was statistically analyzed (Table 2). The soybean high-density genetic map contains 6297 SLAF markers, with a total map distance of 2945.26 cM and an average map distance of 0.47 cM. The number of markers on each chromosome ranges from 131 to 625, the map distance ranges from 122.39 to 192.55 cM, the average map distance ranges from 0.24 to 1.37 cM, the ratio of spacing < 5 cM ranges from 97.73 to 100%, and the maximum spacing ranges from 4 to 14.34 cM.

**Table 2:** Basic information statistics of high-density genetic map

Linkage group	Marker (No)	Map distance (cM)	Average map distance (cM)	Gaps < 5 cM (%)	Max gap (cM)
Gm01	348	145.08	0.42	100.00	4.54
Gm02	372	156.70	0.42	99.46	5.56
Gm03	365	167.92	0.46	99.18	7.46
Gm04	309	140.07	0.45	99.03	7.28
Gm05	183	160.41	0.88	98.35	5.38
Gm06	211	145.65	0.69	99.05	8.32
Gm07	371	161.22	0.44	99.46	9.49
Gm08	255	125.88	0.50	100.00	4.00
Gm09	357	133.83	0.38	99.72	5.50
Gm10	455	123.76	0.27	99.56	7.94
Gm11	131	122.39	0.94	96.92	10.95
Gm12	133	128.10	0.97	97.73	14.34
Gm13	243	125.73	0.52	99.17	10.69
Gm14	224	148.46	0.67	99.55	11.70
Gm15	625	167.77	0.27	99.36	7.90
Gm16	390	192.55	0.49	99.23	7.58
Gm17	345	148.67	0.43	98.84	6.76
Gm18	283	142.60	0.51	100.00	4.43
Gm19	484	152.16	0.32	99.79	6.31
Gm20	213	156.31	0.74	99.06	5.82
Total	6297	2,945.26	0.47	99.17	14.34

**Fig. 3:**
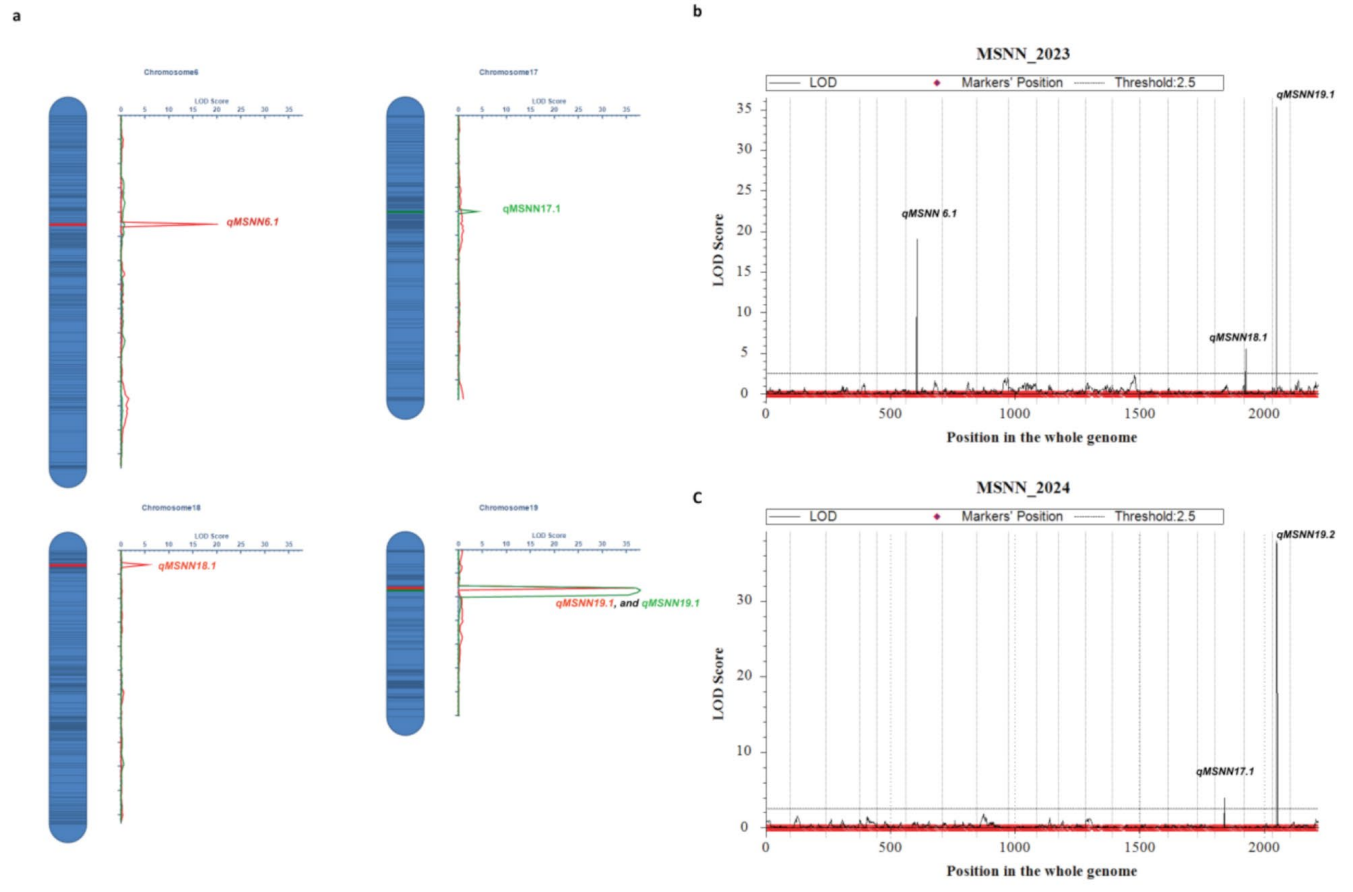
The QTLs of main stem node number traits identified in the RIL-F_2:6_ population using a high-density genetic map. LOD score: 2023, Red lines and 2024, green lines. The x-axis indicated genetic position (cM); the y-axis indicated LOD score

Quantitative trait locus mapping revealed five significant loci controlling MSNN across two growing seasons (Tables 3**and** Fig. 3a-c). In 2023, we detected three QTLs (*qMSNN6.1*, *qMSNN18.1*, and *qMSNN19.1*) distributed on chromosomes 6, 18, and 19. *qMSNN6.1*, flanked by Marker777950 and Marker766908, explained 7.73% of the phenotypic variation with a LOD score of 7.85. This QTL spanned a physical distance of 5.54 Mbp and showed a negative additive effect (-1.27), indicating the allele from ‘Dongsheng 16’ increased MSNN. On chromosome 18, *qMSNN18.1* was mapped between Marker4165022 and Marker4228378, accounting for 3.80% of the phenotypic variation. The most significant QTL detected in 2023 was *qMSNN19.1*, which explained 24.56% of the phenotypic variation with the highest LOD score (22.59) and showed a positive additive effect (2.21).

The 2024 field trial identified two novel QTLs: *qMSNN17.1* on chromosome 17 and *qMSNN19.2* on chromosome 19. *qMSNN17.1*, positioned between Marker5907974 and Marker5935243, explained 3.45% of the phenotypic variation with a negative additive effect (-0.35). Notably, *qMSNN19.2* emerged as the most significant QTL across both years, explaining 43.58% of the phenotypic variation with a LOD score of 37.92. This major QTL spanned a 1.12 Mbp region between Marker1978745 and Marker1711920 and demonstrated a positive additive effect (1.24), suggesting the allele from ‘Qihuang 34’ contributed to increased MSNN. Interestingly, chromosome 19 harbored two major QTLs (*qMSNN19.1* and *qMSNN19.2*) detected in different years, highlighting this genomic region’s significance in regulating MSNN. The identification of these QTLs provides valuable targets for marker-assisted breeding and further investigation of the genetic mechanisms controlling MSNN in soybean.

**Table 3:** QTL mapping analysis of main stem node number in soybean

Year	Chr	QTL names	Position	Left Marker	Right Marker	LOD	PVE(%)	Add	Physical length	Physical position of markers (bp)
2023	6	*qMSNN6.1*	45	Marker777950	Marker766908	7.85	7.73	-1.27	5.54 Mbp	35553901.. 30012890
2023	18	*qMSNN18.1*	6	Marker4165022	Marker4228378	5.49	3.80	-0.75	64.58 kbp	56313709.. 56248879
2023	19	*qMSNN19.1*	16	Marker1939109	Marker1978745	22.59	24.56	2.21	101.67 kbp	45866819.. 45968486
2024	17	*qMSNN17.1*	40	Marker5907974	Marker5935243	3.89	3.45	-0.35	2.84 Mbp	36163447.. 33325892
2024	19	*qMSNN19.2*	17	Marker1978745	Marker1711920	37.92	43.58	1.24	1.12 Mbp	45968486.. 44850990

### Candidate gene analysis within the stable QTL *qMSNN19.2*

The stable QTL *qMSNN19.2* was subjected to detailed analysis to identify potential candidate genes controlling MSNN. Based on the physical position of this QTL, we identified 64 genes within the region. Further screening and functional annotation analysis revealed six prime candidate genes with potential roles in MSNN regulation (Table 4). These genes exhibit diverse molecular functions and biological processes crucial for plant development: Glyma.19G191600 and Glyma.19G193100 both encode serine/threonine protein kinases, with Glyma.19G191600 (CTR1) particularly notable for its involvement in stem cell division regulation and hormone signaling pathways. CTR1 functions in multiple processes including gibberellin biosynthesis and ethylene signaling, suggesting a central role in coordinating hormonal control of stem development. Glyma.19G194800, encoding a cell division protein FtsZ homolog 2 − 1, plays essential roles in cellular organization and division processes. The protein’s GTPase activity and involvement in chloroplast-related functions suggest its potential influence on cellular division patterns during stem development. Glyma.19G195300 codes for a kinesin-like protein KIN-5 C, which participates in microtubule-based processes and spindle organization. Its motor protein activity implies a role in cellular organization during stem development. Glyma.19G196000, encoding a probable UDP-N-acetylglucosamine–peptide N-acetylglucosaminyltransferase SPINDLY, is particularly interesting due to its involvement in both gibberellic acid and cytokinin signaling pathways, as well as its role in cell differentiation. Glyma.19G196300, an mRNA-decapping enzyme-like protein, suggests regulation at the post-transcriptional level, potentially modulating the expression of genes involved in stem development through mRNA processing and degradation. The identification of these candidates, particularly those involved in hormone signaling and cell division, provides valuable insights into the genetic architecture of MSNN in soybean.

**Table 4:** Functional annotations of six potential candidate genes for MSNN

ID	GO	KEGG	PFAM	Description	GO annotation for biological progress
Glyma.19G191600	GO:0006468 GO:0004672 GO:0005524 GO:0004713	K14510	PF07714 PF14381	Serine/threonine-protein kinase CTR1	Regulation of stem cell division; response to fructose; negative regulation of ethylene-activated signaling pathway; gibberellin biosynthetic process; protein serine/threonine kinase activity
Glyma.19G193100	GO:0006468 GO:0005524 GO:0004713 GO:0004672		PF00069 PF07714	Serine/threonine-protein kinase KIPK1	Cytoplasm; nucleus; plasma membrane protein kinase binding; protein serine/threonine kinase activity
Glyma.19G194800	GO:0051258 GO:0003924 GO:0043234 GO:0005525	K03531	PF00091 PF12327	Cell division protein ftsz homolog 2 − 1	Cytoplasm; chloroplast fission; chloroplast stroma; protein self-association; chloroplast thylakoid; GTPase activity; GTP binding; chloroplast thylakoid membrane; chloroplast
Glyma.19G195300	GO:0005524 GO:0005871 GO:0007018 GO:0008017 GO:0003777	K10398	PF00225	Kinesin-like protein KIN-5 C	Cytoplasm; spindle; microtubule motor activity; microtubule-based movement; microtubule binding; microtubule; ATP binding
Glyma.19G196000	GO:0005515		PF00515 PF13424 PF13844 PF13414	Probable UDP-N-acetylglucosamine–peptide N-acetylglucosaminyltransferase SPINDLY	Negative regulation of gibberellic acid mediated signaling pathway; cytoplasm; gibberellic acid mediated signaling pathway; cytokinin- activated signaling pathway; protein glycosylation; cell differentiation; cytosol; nucleus
Glyma.19G196300		K12611	PF06058	mRNA- decapping enzyme-like protein	mRNA binding; deadenylation-dependent decapping of nuclear-transcribed mRNA; enzyme activator activity; deadenylation-independent; decapping of nuclear-transcribed mRNA; Cytoplasm; mRNA processing; P-body; hydrolase activity;

### Gene ontology (GO) and KEGG pathway analysis

**Fig. 4:**
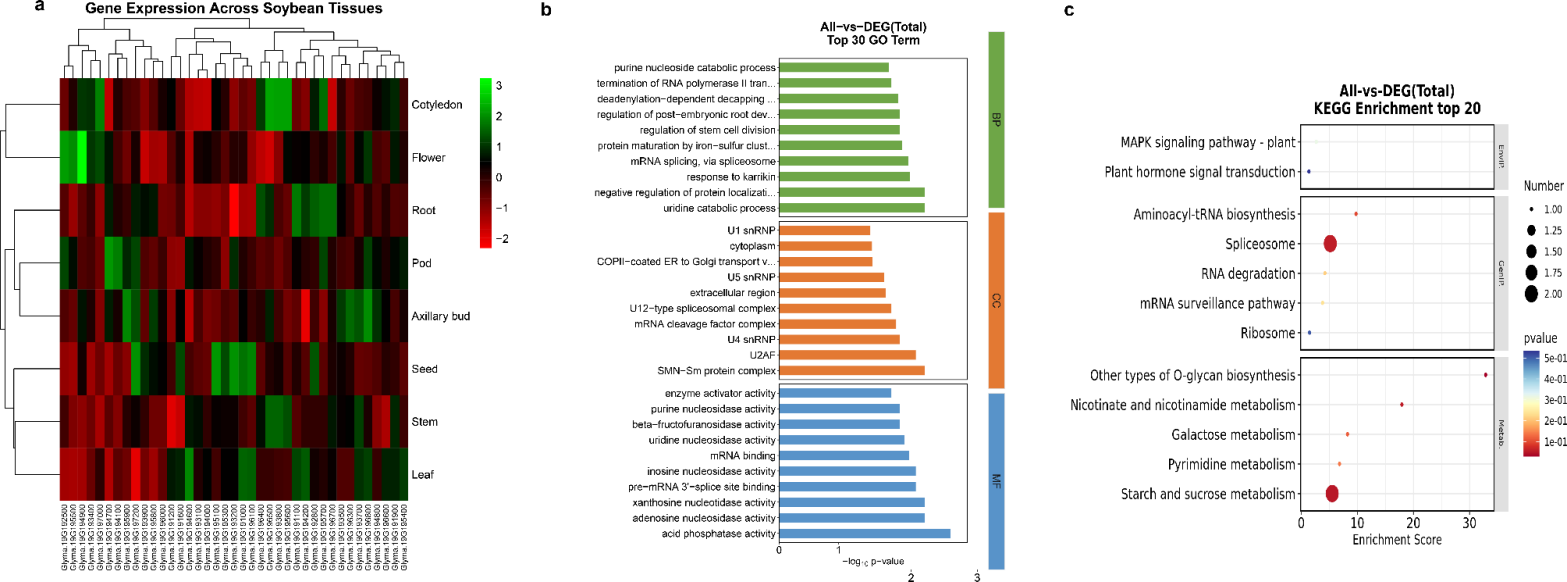
Enrichment analysis of candidate genes on GO terms and KEGG pathways. (**a**) The expression pattern of the identified candidate genes (**b**) GO term enrichment analysis. Al pair, showing the top 30 terms. (**c**) KEGG pathway enrichment analysis of the. Al pair, showing the top 20 pathways. Count represents the number of genes associated with the specific function or pathway. The larger the red circle, the greater the number; conversely, the smaller the circle, the fewer the genes. -related genes

The expression pattern of the identified candidate genes was investigated in Williams 82 cv. using the RNA-seq data available at SoyBase. The dataset includes several plant tissues, including leaves, nodules, roots, pods, and seeds (Fig. 4a). Thirty-nine of the 64 identified genes have available RNA-seq data. Then these genes were used to conduct pathway analysis in the GO and Kyoto Encyclopedia of Genes and Genomes (KEGG) databases (Fig. 4b-c),** which** revealed coordinated regulation of many biological processes and pathways. GO enrichment analysis found significant terms in biological processes (BP), cellular components (CC), and molecular activities. Biology highlighted RNA processing pathways, with “mRNA splicing, via spliceosome” being particularly significant (-log10 *p*-value > 2). This was accompanied by enrichment of “deadenylation-dependent decapping” and “termination of RNA polymerase II transcription.” Splicing-related complexes such U1, U2AF, U4, and U5 snRNPs and the SMN-Sm protein complex were enriched in the cellular component analysis, supporting our findings. This pattern was supported by molecular function words that enriched RNA-binding and nucleosidase functions. KEGG pathway analysis illuminated molecular processes. The research identified genetic, metabolic, and environmental information processing functional groups. The spliceosome pathway demonstrated the highest enrichment score (∼ 8, *p*-value < 0.1), supporting GO analysis results. Additionally, aminoacyl-tRNA production, RNA degradation, and mRNA monitoring pathways were considerably enriched. Significant metabolic pathway enrichment, especially starch and sucrose metabolism (enrichment score ∼ 8). Additionally, O-glycan biosynthesis, nicotinate and nicotinamide metabolism, galactose metabolism, and pyrimidine metabolism were enriched, suggesting extensive metabolic reprogramming. Despite modest enrichment, MAPK signaling and plant hormone signal transduction were statistically significant.

### Differential expression analysis between DS16 and QH34 lines

**Fig. 5:**
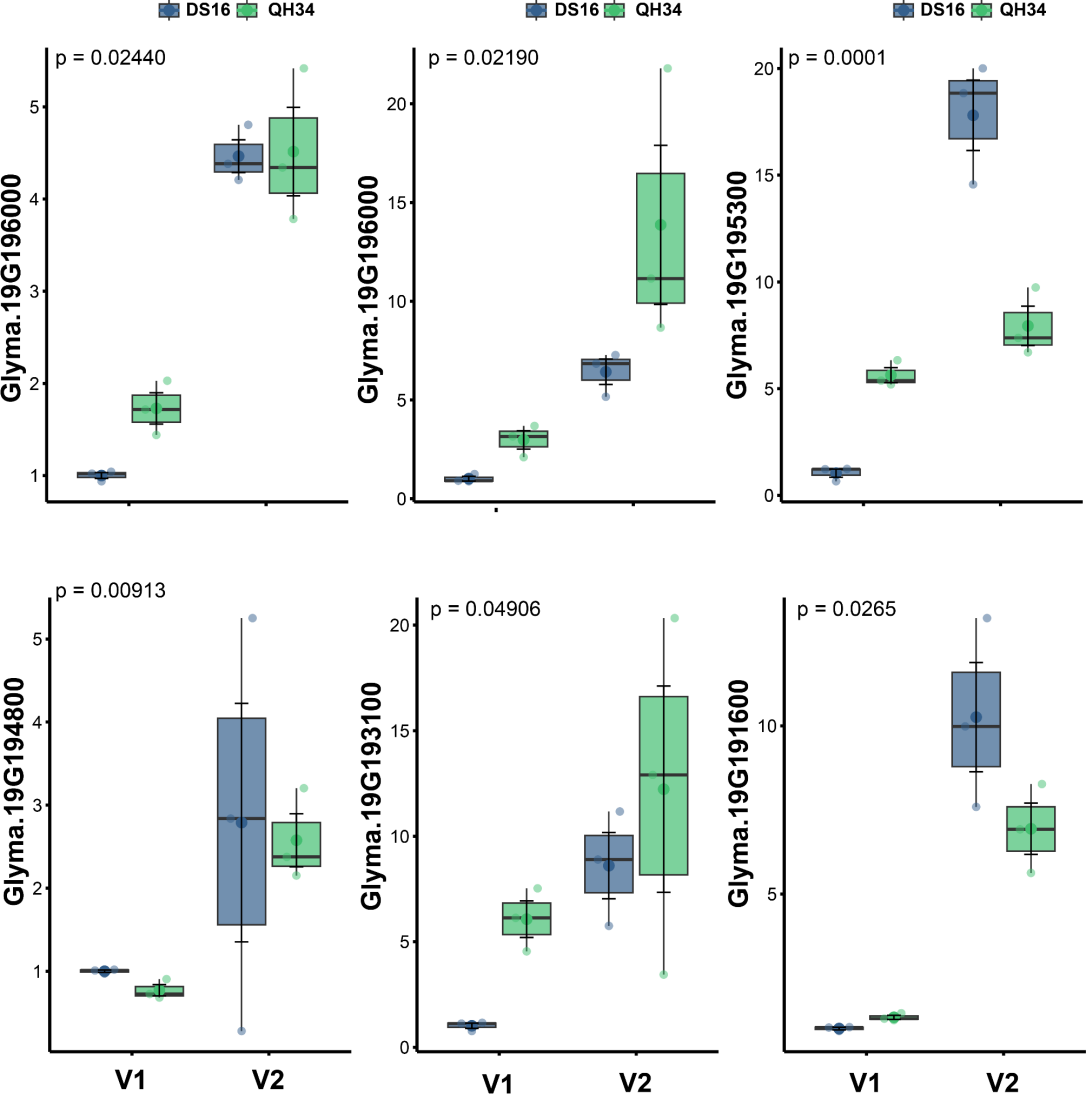
Differential expression analysis of six candidate genes between DS16 and QH34 soybean during V1 and V2 developmental stages

Comparative analysis revealed significant differential expression patterns between DS16 and QH34 lines across six genes during developmental stages V1 and V2 (Fig. 5). The most pronounced difference was observed in Glyma.19G195300, which exhibited highly significant differential expression (*p* = 0.0001) between the two lines. In DS16, this gene showed markedly elevated expression levels at the V2 stage compared to both its V1 expression and the QH34 line at either stage. Glyma.19G196000 demonstrated significant variation between the lines (*p* = 0.02440), with QH34 showing higher expression levels at both developmental stages. Notably, both lines exhibited increased expression from V1 to V2, though the magnitude of increase was more pronounced in QH34. Similarly, Glyma.19G196000 displayed significant differential expression (*p* = 0.02190), with QH34 maintaining consistently higher expression levels, particularly at the V2 stage. Glyma.19G194800 exhibited an interesting inverse pattern (*p* = 0.00913), where DS16 showed higher expression levels at V2 compared to QH34, contrasting with the V1 stage where expression levels were comparable between lines. Glyma.19G193100 demonstrated significant differences (*p* = 0.04906) with both lines showing increased expression at V2, though QH34 exhibited greater variability in expression levels. Glyma.19G191600 analysis revealed significant differences between the lines (*p* = 0.0265), with DS16 showing notably higher expression at V2 compared to QH34. This pattern was distinct from the V1 stage, where expression levels were relatively similar between lines. Collectively, these results indicate a complex regulatory pattern across these six genes, with most showing stage-specific differential expression between DS16 and QH34 lines. The consistent statistical significance across all genes (*p* < 0.05) suggests a coordinated genetic response that differs between the two lines during early vegetative development.

### KASP marker development and identification of SNPs associated with MSNN traits

**Fig. 6:**
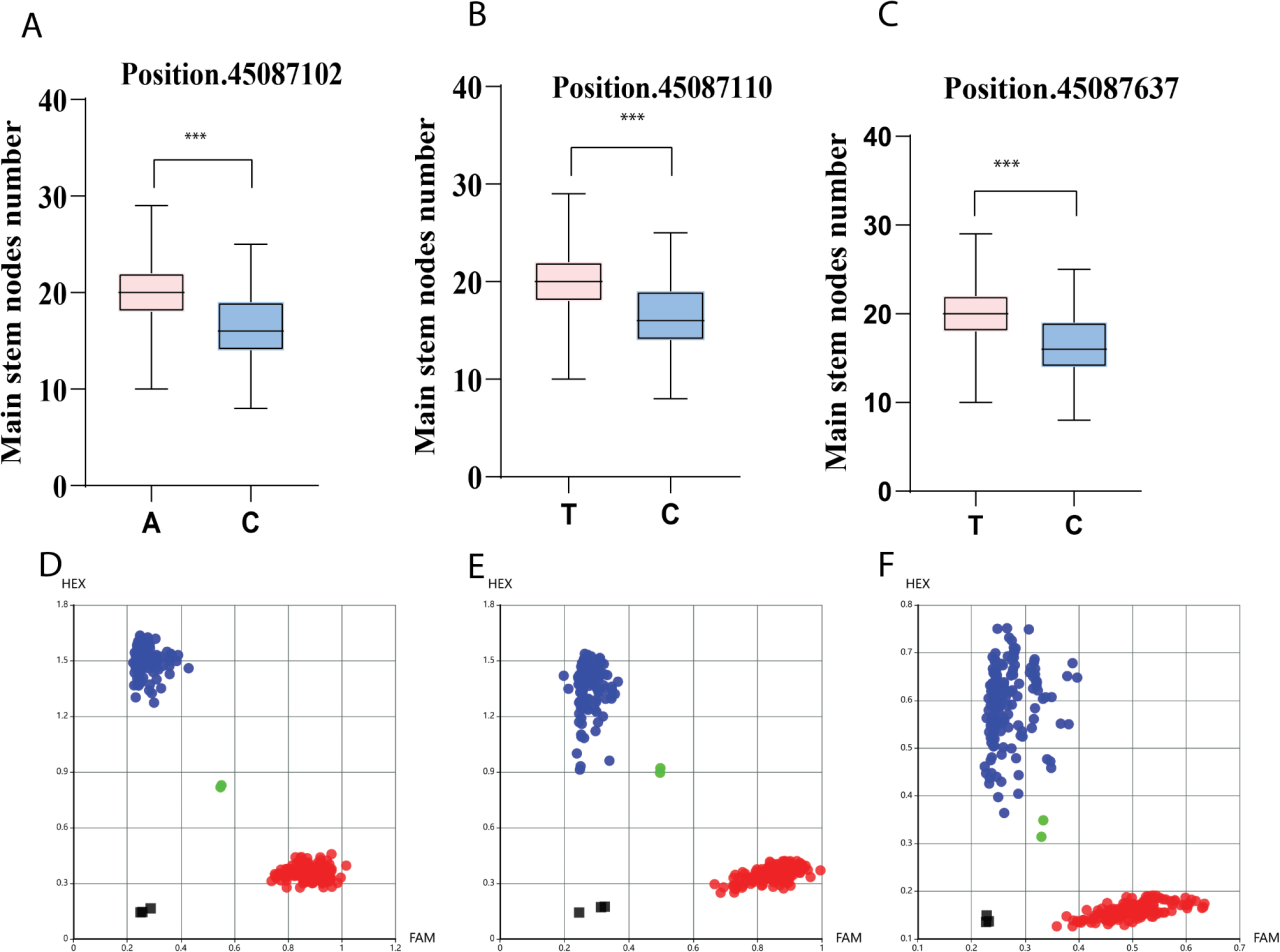
Kompetitive allele-specific PCR (KASP) verification of a significant single-nucleotide polymorphism (SNP) related to the MSNN. (**A**, **B**, and **C**) The variance of positions 45,087,102, 45,087,110, and 45,087,637 for accessions with different alleles; (**D**, **E**, and **F**) Scatter plots of KASP markers for positions 45,087,102, 45,087,110, and 45,087,637, respectively; red dots and blue represent the homozygous alleles, green represent heterozygous alleles, and black squares on the bottom left of the plot indicate the no-template control; ***significant difference at *p* < 0.001

**Fig. 7:**
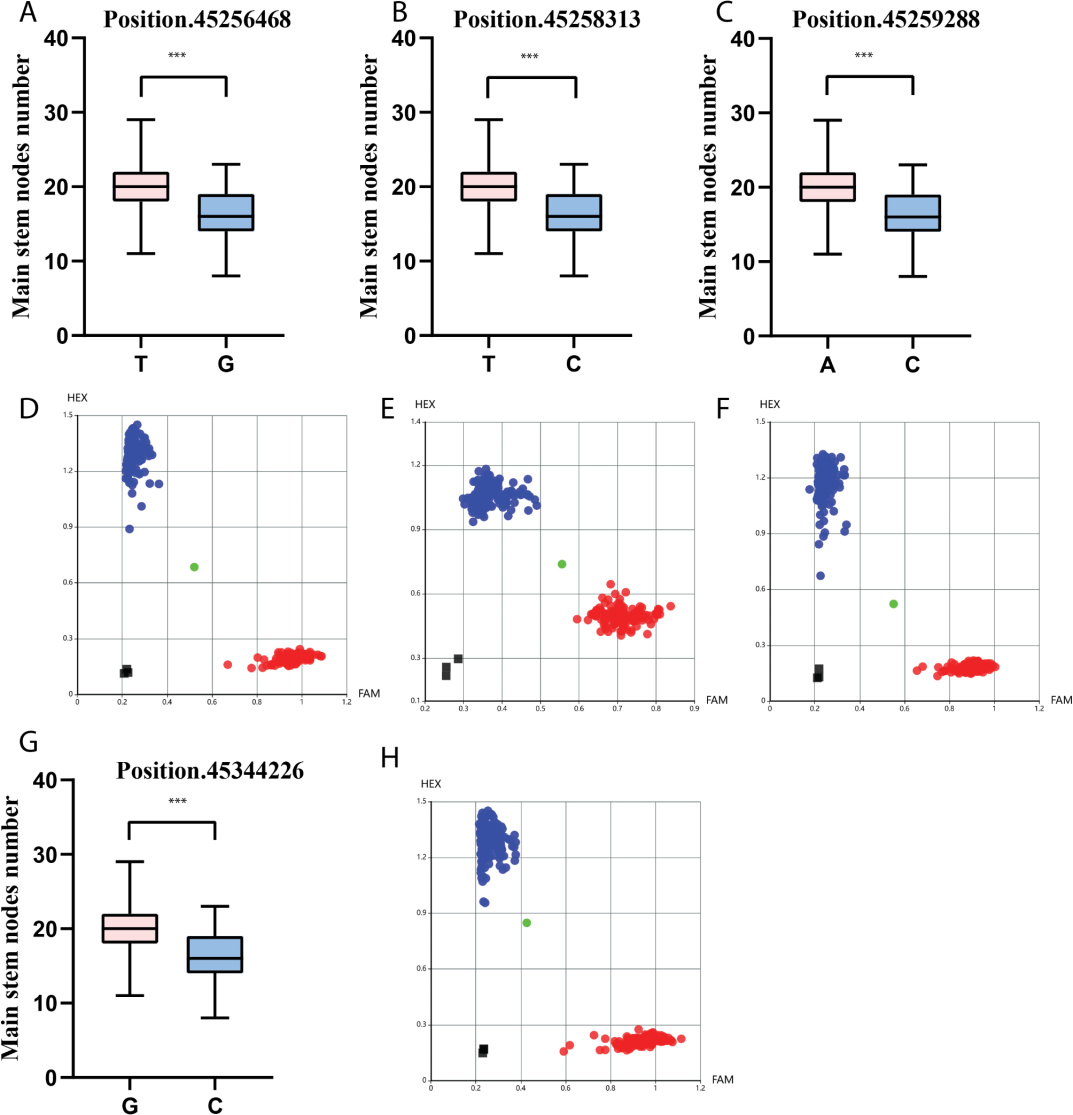
Analysis of the Kompetitive Allele Specific PCR (KASP). Scatter plots show the selected KASP assays clustering of varieties on the X-(VIC) and Y-(FAM) axes. The red dot represents heterozygous genotype and blue and green dots represent the homozygous. phenotype and genotype analysis for Glyma.19G195300, three SNPs were found at positions 45,256,468(**A**, **D**), 45,258,313(**B**, **E**), and 45,259,288 (**C**, **F**). Also, Glyma.19G196300, SNP at position 45,344,226 (**G**-**H**); ***significant difference at *p* < 0.001

To enhance the application of major QTLs in soybean breeding, we developed KASP markers targeting key candidate genes associated with MSNN. Significant single nucleotide polymorphisms (SNPs) were identified in three candidate genes: *Glyma.19G193100*, *Glyma.19G195300*, and *Glyma.19G196300* (Table 5), each exhibiting notable associations with MSNN traits. In Glyma.19G193100, we detected three SNPs at positions 45,087,102, 45,087,110, and 45,087,637. The A allele at position 45,087,102 (Fig. 6A-D) and the T allele at position 45,087,110 (Fig. 6B-E) were associated with reduced MSNN compared to their respective C alleles Similarly, the T allele at position 45,087,637 (Fig. 6C-F) was also linked to lower MSNN. These findings suggest that specific alleles at these loci may negatively influence MSNN in soybean. For Glyma.19G195300, three SNPs were identified at positions 45,256,468, 45,258,313, and 45,259,288. The T allele at position 45,256,468 (Fig. 7A-D), the T allele at position 45,258,313 (Fig. 6B-E), and the A allele at position 45,259,288 (Fig. 7C-F) were all positively correlated with increased MSNN compared to their respective G and C alleles. In Glyma.19G196300, a single SNP at position 45,344,226 (Fig. 7G-H) revealed that the G allele was associated with higher MSNN compared to the C allele. Collectively, these findings demonstrate that specific SNP variations within these candidate genes significantly influence MSNN traits in soybean.

**Table 5:** SNP information of the kompetitive allele specific PCR (KASP)

ID	Chr	Pos	ref	DS16	QH34	Codon_change
Glyma.19G193100	19	45,087,102	A	A	C	Tct/Gct
		45,087,110	T	T	C	aAt/aGt
		45,087,637	A	T	A	gaT/gaA
Glyma.19G195300	19	45,256,468	G	T	G	ttG/ttT
		45,258,313	C	T	C	Cca/Tca
		45,259,288	C	A	C	tCt/tAt
Glyma.19G196300	19	45,344,226	C	G	C	atC/atG

## Discussion

QTL mapping is a powerful tool for analyzing quantitative traits in soybean, and the quality of the underlying genetic map significantly influences the resolution of QTL detection. High-density linkage maps are especially important for QTL mapping, positional cloning, comparative genomics, MAS, and identification of functional genes [40, 41]. Currently, although SNPs have become the first-selected genetic markers for high-density linkage map construction, their widespread application remains limited by the high costs of high-throughput sequencing and genotyping [42]. Fortunately, NGS-based marker identification and genotyping technologies provide a powerful method for the identification of large numbers of SNPs. The SLAF-seq approach balances genotyping accuracy and sequencing cost, thus providing an economical and efficient method for linkage mapping of non-model species with complex genomes [35, 43]. As a result, the construction of a high-density genetic map is crucial for improving the precision and reliability of QTL mapping in soybean. This enhanced resolution not only aids in QTL identification but also holds great potential for applications in molecular marker-assisted selection [33, 37]. SLAF-seq technology has been extensively utilized for constructing genetic maps in genetic research [34, 36, 44, 45]. Li et al. [35] utilized SLAF-seq to develop a high-density genetic map in soybean, which comprises 3541 SLAF markers and has an average genetic distance of 0.72 cM. Cao et al. [37] developed a soybean genetic map that includes 3255 SLAF markers, exhibiting an average genetic distance of 0.66 cM. Enhancing the marker density has the potential to elevate the resolution of genetic maps, along with improving the accuracy and quality of QTL mapping [37, 46–48]. In this study, a map included 6,297 SLAF markers, covering 2,945.26 cM with an average marker distance of 0.47 cM was developed. The distribution of markers across chromosomes ranged from 131 to 625, with map distances varying between 122.39 and 192.55 cM. The ratio of intervals less than 5 cM ranged from 97.73 to 100%, and the maximum interval distance was between 4 and 14.34 cM. The high-density soybean genetic map is likely attributable to the substantial population size, the augmented number of SLAF markers, and the reduced gaps between markers, indicating that our genetic map serves as a valuable reference for soybean and can facilitate further genetic mapping and the prediction of MSNN-related genes.

MSNN is a critical agronomic trait influencing yield numerous studies on soybean have identified QTLs for MSNN on various chromosomes with diverse plant materials [14, 17, 21, 24]. we identified novel QTLs linked to MSNN in soybean by SLAF-seq and predicted candidate genes inside these QTLs utilizing high-density genetic maps, which may facilitate the identification of new QTLs for MSNN. In this study, five QTLs linked to MSNN were identified, with three detected in 2023 and two in 2024. These QTLs were located on chromosomes 6, 17, 18, and 19 (*qMSNN6.1*, *qMSNN17.1*, *qMSNN18.1*, and *qMSNN19.2*). The LOD scores ranged from 3.8 to 37.9, and the phenotypic variance explained (PVE) ranged from 3.45 to 43.58%. Notably, *qMSNN19.2* on chromosome 19 showed the highest LOD (37.9) and PVE (43.58%) in 2024, with markers Marker1978745 and Marker1711920 spanning 1.117 Mb. In 2023, *qMSNN19.1* on chromosome 19 had a LOD of 22.59 and PVE of 24.56%, with markers Marker1939109 and Marker1978745 separated by 101.43 kb. To date, SoyBase (http://www.soybase.org) has documented 38 MSNN QTLs identified through linkage mapping, along with an additional 45 MSN QTLs reported in various studies using genome-wide association studies (GWAS) [14, 16, 49]. Fu et al. [17] used RTM-GWAS to identify 76 MSN QTLs with 183 alleles, collectively explaining 65.63% of the phenotypic variance (PV) in a Northeast China soybean germplasm population comprising 306 varieties. Similarly, Fahim et al. [18] applied RTM-GWAS to detect 151 MSN QTLs with 587 alleles, accounting for 90.64% of the PV, including both main-effect loci and QTL-by-environment interaction (QEI) loci, in a Chinese cultivated soybean population of 821 accessions. In previous studies, several MSNN-related QTLs have been identified. For example, qlNN-2-1 was found on chromosome 2 within the genomic interval of 29,959,409–41,608,316 bp [20]. Similarly, *qlNN-5-1* was identified on chromosome 5 within the interval of 22,088,622–41,360,809 bp [21]. On chromosome 6, *qlNN-6-2* was detected in the interval of 11,860,267–12,150,538 bp [50]. Additionally, *qlNN-13-1* (*qlRDNN-13-1*) was identified on chromosome 13 within the interval of 444,838–43,052,819 bp [22, 51]. On chromosome 17, *qlNN-17-2* was found in the interval of 7,296,590 − 9,660,500 bp [23]. Furthermore, *qnNN-5-4* (37,951,491 bp) and *qnRDNN-5-4* (38,349,709 bp) were identified on chromosome 5 [21].

Only a few number of genes have been directly associated with MSNN across various crops. A novel *ricMT* gene exhibited high expression levels in stem nodes *ZmMADS3* expression was observed in the stem nodes of maize, and transgenic maize exhibited a reduction in the number of nodes [52]. *Dt1* regulated the number of nodes in soybean by influencing stem growth habit [53]. Thus, exploring potential candidate genes for MSNN holds substantial significance. The detailed analysis of the *qMSNN19.2* region led to the identification of 64 genes, from which six candidates were selected based on their functional annotations. Particularly noteworthy is *Glyma.19G191600* (CTR1), which integrates multiple hormone signaling pathways including ethylene and gibberellin responses. The involvement of *Glyma.19G196000* (SPINDLY) in both gibberellic acid and cytokinin signaling suggests hormone-mediated regulation of MSNN development. These findings align with previous research demonstrating the critical role of plant hormones in controlling stem architecture and node development [54–56]. The identification of cell division-related genes such as *Glyma.19G194800* (FtsZ homolog 2 − 1) and cytoskeletal proteins like *Glyma.19G195300* (KIN-5 C) suggests that MSNN development is regulated through complex cellular mechanisms involving both hormonal signals and structural components [57, 58]. The presence of regulatory proteins such as KIPK1 (*Glyma.19G193100*) and an mRNA-decapping enzyme (*Glyma.19G196300*) indicates multiple levels of control in MSNN determination.

The identification of SNPs in key candidate genes associated with MSNN in soybean offers valuable insights into the genetic regulation of plant architecture and its potential applications in breeding. In this study, we developed KASP markers for three genes *Glyma.19G193100*, *Glyma.19G195300*, and *Glyma.19G196300*—that exhibit significant associations with MSNN traits, providing markers for marker-assisted selection (MAS) to improve soybean yield through enhanced node number. The SNPs identified in this study within *Glyma.19G193100*, *Glyma.19G195300*, and *Glyma.19G196300* contribute to our understanding of the molecular mechanisms controlling MSNN in soybean, with implications for optimizing yield. The gene *Glyma.19G193100* is associated with reduced MSNN when specific alleles (A at 45087102 and T at 45087110) are present, suggesting that the expression of this gene may play a role in limiting node formation. This is consistent with findings from other studies that identified genes involved in the regulation of branching and node development in legumes [20]. The negative correlation between these alleles and MSNN suggests that their inclusion in breeding programs may be beneficial for controlling excessive node formation, which can lead to competition for resources and reduce overall plant productivity [9]. Conversely, the SNPs in *Glyma.19G195300* and *Glyma.19G196300* were associated with increased MSNN, suggesting that these genes could be targeted to enhance node number in breeding programs. The positive correlation between the T allele at 45,256,468 and 45,258,313 in *Glyma.19G195300* with MSNN highlights the potential of these alleles to boost node count and improve yield. Similarly, the G allele at 45,344,226 in *Glyma.19G196300* was found to promote increased node number, offering another potential target for breeding. These results align with earlier research that identified genes regulating stem growth and node production in legumes, which are crucial for improving yield [36, 47, 51]. The development of KASP markers for these SNPs offers a promising tool for marker-assisted selection in soybean breeding programs. These findings contribute to our understanding of the genetic control of soybean architecture and provide a foundation for future efforts aimed at enhancing soybean productivity through molecular breeding.

## Conclusion

This study explored the genetic basis of MSNN, a key yield-related trait in soybean, using QTL mapping and candidate gene analysis in a population of 325 recombinant inbred lines (RILs). Significant transgressive segregation and continuous variation in MSNN highlighted its complex genetic architecture. A high-density genetic map constructed with 6,297 SLAF markers identified five significant QTLs, with *qMSNN19.2* on chromosome 19 showing the highest LOD (37.9) and PVE (43.58%). Further analysis narrowed down six candidate genes within the *qMSNN19.2* region, including proteins involved in kinase activity, cell division, and mRNA decapping. These findings emphasize the roles of these genes in regulating MSNN and pave the way for KASP marker development to assist soybean breeding programs. Overall, the insights from this study provide valuable resources for improving soybean cultivars through enhanced MSNN traits.

## Electronic supplementary material

Below is the link to the electronic supplementary material.

Supplementary Material 1

## Data Availability

All data generated or analyzed during this study are included in this published article and its supplementary information files. The datasets used and/or analyzed during the current study are available from the corresponding author (Honglei Ren) on reasonable request.
