# Supplementary material for: Integrative QTL mapping and candidate gene analysis for main stem node number in soybean
Source: BMC Plant Biol. 2025 Apr 3;25:422. doi: 10.1186/s12870-025-06457-2 (PMC11967112; doi:10.1186/s12870-025-06457-2)
Supplement: Supplementary file 1 — Supplementary Material 1 [file 12870_2025_6457_MOESM1_ESM.docx]

**Table S1.** Primer sequences of Quantitative real-time PCR (qRT-PCR)

| Primer name | Sequence |
| --- | --- |
| Glyma.19G191600-F | CAATGAACCCTGGAAACGCC |
| Glyma.19G191600-R | TACAACTGTGCCTCTGGGAC |
| Glyma.19G193100-F | CAAAGCAATCAGTGCCTGCC |
| Glyma.19G193100-R | ATGCCATTTGAAACCCTGATATG |
| Glyma.19G194800-F | ACCTATGCAGGCCAGTCAAC |
| Glyma.19G194800-R | TGCCAACAAGCCTCAACTCA |
| Glyma.19G195300-F | CGACAAATGGATCTGGTAGACG |
| Glyma.19G195300-R | GCTGCATGAGCACCTCCAT |
| Glyma.19G196000-F | TATAGCGAGGCAAAGGTGGG |
| Glyma.19G196000-R | AAATCGGGGAAGGGAGGAGA |
| Glyma.19G196300-F | TTTGCTTGGAGTGAGTGCGA |
| Glyma.19G196300-R | CTAGCTCGCTCAAACCCTCC |

Table S2. Number distribution of high quality polymorphic SLAF on each chromosome

| ChrID | SLAF Number | ChrID | SLAF Number |
| --- | --- | --- | --- |
| Gm01 | 349 | Gm12 | 134 |
| Gm02 | 388 | Gm13 | 244 |
| Gm03 | 366 | Gm14 | 225 |
| Gm04 | 310 | Gm15 | 626 |
| Gm05 | 184 | Gm16 | 570 |
| Gm06 | 212 | Gm17 | 346 |
| Gm07 | 372 | Gm18 | 284 |
| Gm08 | 256 | Gm19 | 690 |
| Gm09 | 358 | Gm20 | 214 |
| Gm10 | 457 | Other | 0 |
| Gm11 | 132 | Total | 6,717 |

**Table S3.** Primer sequences of Kompetitive allele-specific PCR (KASP) markers.

| Primer name | Sequence |
| --- | --- |
| Chr19-45087102Fa | GAAGGTGACCAAGTTCATGCTCAAACCAGGTTTATTTGAACCAGA |
| Chr19-45087102Fc | GAAGGTCGGAGTCAACGGATTAAACCAGGTTTATTTGAACCAGC |
| Chr19-45087102R | GGCATTAGACTCTACCAGTCCTGA |
| Chr19-45087110Ra | GAAGGTGACCAAGTTCATGCTCCTGAAAACGGAGTAAACTTGAGTAA |
| Chr19-45087110Rg | GAAGGTCGGAGTCAACGGATTCCTGAAAACGGAGTAAACTTGAGTAG |
| Chr19-45087110F | ACTTGGTGTTCTTCACTTTTGCAA |
| Chr19-45087637Fa | GAAGGTGACCAAGTTCATGCTTTTGTATCAATGAAGTCCCAGCA |
| Chr19-45087637Ft | GAAGGTCGGAGTCAACGGATTCTTTGTATCAATGAAGTCCCAGCT |
| Chr19-45087637R | TCTCAATCTTCCCAAATTGCATT |
| Chr 19-45256468Rc | GAAGGTGACCAAGTTCATGCTAGAAAAGAATAACACCTACCAATTTTC |
| Chr 19-45256468Ra | GAAGGTCGGAGTCAACGGATTGAGAAAAGAATAACACCTACCAATTTTA |
| Chr 19-45256468F | AGCTCATCAGGATAATGCTTCG |
| Chr 19-45258313Fc | GAAGGTGACCAAGTTCATGCTCCAAACATATTTCAGCAACAGAGC |
| Chr 19-45258313Ft | GAAGGTCGGAGTCAACGGATTACCAAACATATTTCAGCAACAGAGT |
| Chr 19-45258313R | TAGTCATTGACAATCAGTTTGCCAC |
| Chr 19-45259288Rg | GAAGGTGACCAAGTTCATGCTTGGTTTCAACTCCTTTACATCATTAG |
| Chr 19-45259288Rt | GAAGGTCGGAGTCAACGGATTTGGTTTCAACTCCTTTACATCATTAT |
| Chr 19-45259288F | AGGGGACAATAGAGTCTCTTCGA |
| Chr 19-45344226Fc | GAAGGTGACCAAGTTCATGCTGGAACTTCAGCCAGTGTCAATTATC |
| Chr 19-45344226Fg | GAAGGTCGGAGTCAACGGATTGGAACTTCAGCCAGTGTCAATTATG |
| Chr 19-45344226R | CATGCAACAACATTACGTACGCTT |
